# Diagnostic information Profiling and Evaluation of Causative Fungi of Fungal Keratitis Using High-throughput Internal Transcribed Spacer Sequencing

**DOI:** 10.1038/s41598-020-58245-7

**Published:** 2020-02-03

**Authors:** Zhichao Ren, Qing Liu, Yuqian Wang, Yanling Dong, Yusen Huang

**Affiliations:** 1 School of Medicine and Life Sciences, University of Jinan-Shandong Academy of Medical Sciences, Jinan, China; 2 State Key Laboratory Cultivation Base, Shandong Provincial Key Laboratory of Ophthalmology, Shandong Eye Institute, Shandong First Medical University & Shandong Academy of Medical Sciences, Qingdao, China

**Keywords:** Genetic techniques, Eye diseases, Corneal diseases

## Abstract

Early and accurate diagnosis is essential for the targeted management of fungal keratitis (FK), which is one of the major blinding eye diseases worldwide. To elucidate the diagnostic information of high-throughput internal transcribed spacer (ITS) sequencing for identifying causative fungi of FK, 38 patients who were highly suspected of having FK were included in this research. *In vivo* confocal microscopy, potassium hydroxide smear, and fungal culture were performed to diagnose FK. Culture and ITS sequencing were used to identify causative fungi. We hypothesized that the dominant genus was the result of pathogen identification by ITS sequencing. Thirty-five patients were eventually diagnosed with FK, with fungal pathogens found by confocal microscopy in 27 patients (77.14%), by smear examination in 27 patients (77.14%), by culture in 25 patients (71.43%), and by ITS sequencing in 26 patients (74.29%). Eight causative fungal genera were determined by ITS sequencing, while five causative fungal genera were identified based on the morphology of the cultured pathogens. The results of ITS sequencing and culture were coincident in 10 patients with FK (28.57%). It is concluded that ITS sequencing, to some extent, challenged fungal culture and might be an optional complement in identifying fungal pathogens in corneas.

## Introduction

Fungal keratitis (FK), usually prevailing in agriculture-based geographic regions with tropical and subtropical climates, affects over a million people each year, three quarters of whom will probably lose their eyesight^1,2^. It often causes devastating consequences like severe corneal inflammation, ulcers, scars, and even perforations^1–5^. One of the reasons is the lack of methods for proper identification of causative organisms^1,2^. This kind of infectious disease can be associated with different fungal pathogens, which have varied sensitivities to a series of antifungal drugs, just as varying bacteria have their specific sensitivities to antibiotics. An early and accurate diagnosis and targeted treatment are vital for the prognosis of FK^1,5^.

*In vivo* confocal microscopy (IVCM), corneal scraping smear, and fungal culture are applied widely in the diagnosis of FK^5^, but neither the success rate nor the accuracy of identifying specific pathogens is immensely satisfying. In recent years, PCR with gene chipping has been deemed to be a promising technology in the identification of pathogenic fungi for its high sensitivity and specificity^5^. However, since pathogenic fungi also exist on the normal human ocular surface, this tool sometimes can generate false-positive results^4^.

With the development of DNA sequencing and molecular genetics, high-throughput sequencing of internal transcribed spacer (ITS) genes has become a main research approach to microbial classification^4,6^. Compared with traditional fungal culture, high-throughput ITS sequencing, which is not limited to the environment, time, activity of fungi, and sample size, can produce more information about ocular microbiome. Moreover, ITS sequencing seems to be more affordable with global availability than gene chipping, multi-locus sequencing or nanopore sequencing. This should not be neglected, because the estimated incidence rates of FK are comparatively high in developing countries, and sufferers are basically agricultural workers who have a low socioeconomic status^1^. In this study, we attempted to elucidate the diagnostic information of high-throughput ITS sequencing for identifying causative fungi of FK.

## Materials and Methods

### General information

Our research was approved by the Ethics Committee of Shandong Eye Institute (2019–26) and registered on Chinese Clinical Trial Registry (ChiCTR1900023720). All procedures adhered to the tenets of the Declaration of Helsinki. Informed consent was obtained from all participants.

### Inclusion criteria

Patients were highly suspected of having FK if they met at least two of the following criteria^5,7,8^: ① a history of eye trauma with plants or soil, ② chronic local or systemic use of corticosteroids or antibiotics, ③ corneal ulceration with a dry appearance, ④ corneal ulceration with pseudopods, ⑤ corneal ulceration with satellite lesions, ⑥ corneal ulceration with elevated lesions, ⑦ presence of endothelial plaques, and ⑧ presence of dense hypopyon. A continuous series of 38 patients with suspected FK and no history of contact lens wear in the diseased eye were included in this prospective study.

### Diagnostic criteria

**Figure 1:**
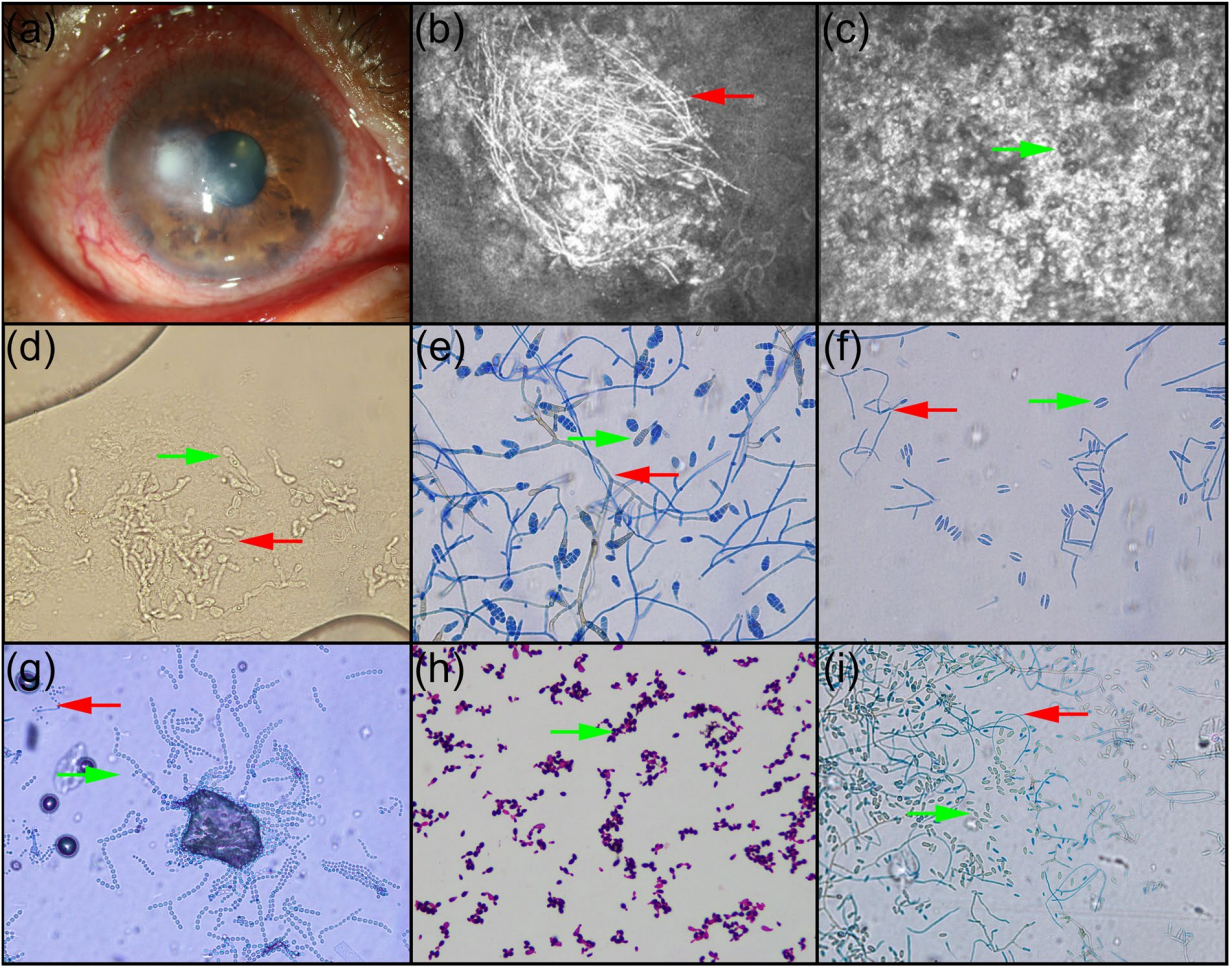
Representative images of the eye with fungal keratitis (**a**), fungal hyphae on IVCM at 800 magnifications (**b**), spores on IVCM at 800 magnifications (**c**), KOH smear at 400 magnifications (**d**), and cultured isolates stained with lactophenol cotton blue stain at 400 magnifications, including *Alternaria* (**e**), *Fusarium* (**f**), *Aspergillus* (**g**), *Saccharomyces* (**h**), and *Acremonium* (**i**). Red arrows to the left refer to hyphae, and green arrows to the right refer to spores.

A diagnosis of FK was confirmed as long as one of the results of IVCM, potassium hydroxide (KOH) smear, and fungal culture was positive as shown in Fig. 1^7,9,10^.

### Sample collection

**Figure 2:**
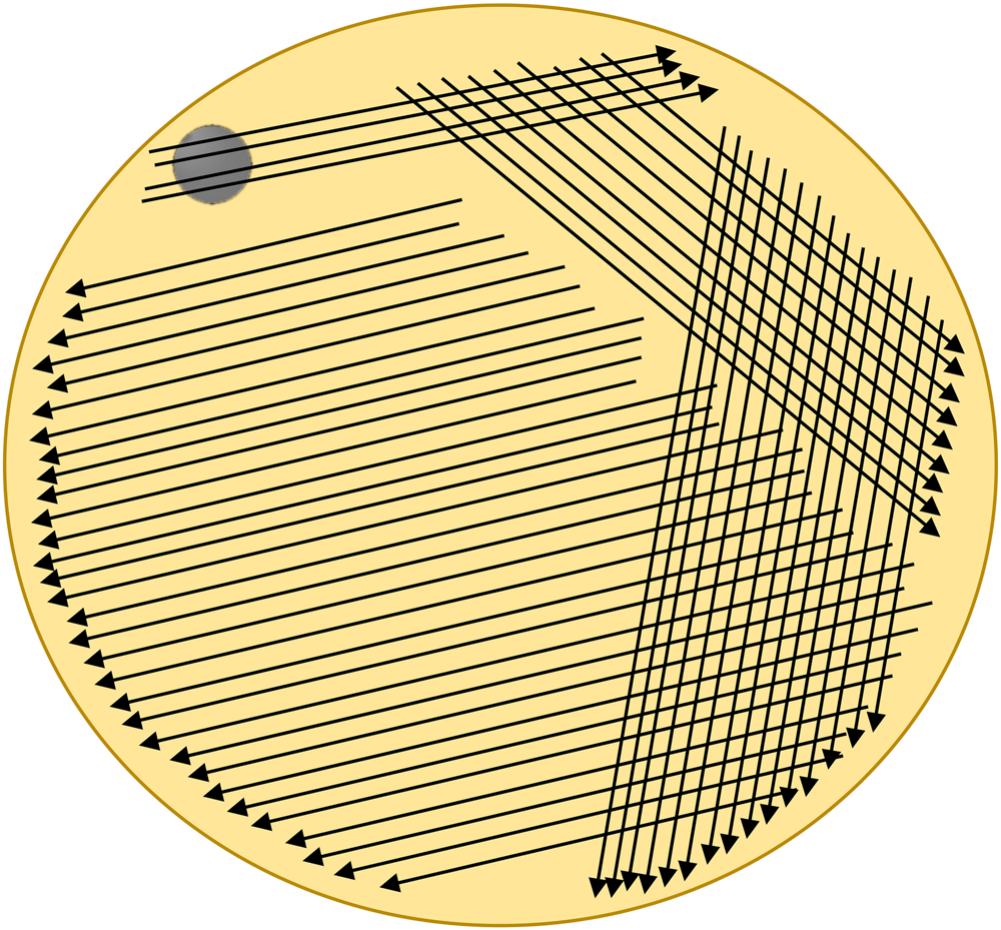
Sample was streaked onto the Sabouraud dextrose agar medium plate along 4 directions. The intersection angle between adjacent areas was approximately 120°, so as to make full use of the whole agar medium. After completing the sample streaking in one area, the inoculation ring was burned to remove remaining fungi, before it was used for streaking in the next area.

The patients lay supine for collection of corneal scraping samples and conjunctival samples in a disinfected room. After eye lids were wiped using Iodophor, the facial area except the eyes was covered with a sterile surgical drape. A drop of oxybuprocaine hydrochloride eye drops was dripped to each diseased eye for anesthesia before sampling^11^. The corneal lesions were scraped using an ophthalmic microsurgical knife (Cat. No. MR-G137A, Suzhou Mingren Medical Equipment Co., Suzhou, China) under a microscope. Then the scrapings were smeared on a sterile glass slide for KOH smear, streaked on Sabouraud dextrose agar medium containing 50 μg/ml chloramphenicol (Cat. No. A0697077, Baibo Biotechnology, Jinan, China) for culture (Fig. 2), and stored in a 1.5-ml sterile and DNase/RNase free microcentrifuge tube (Cat. No. CS015-0041, Excell Biotechnology, Shanghai, China) for ITS sequencing, respectively.

The palpebral conjunctiva, bulbar conjunctiva, and fornical conjunctiva were also rubbed lightly using a sterile swab^12,13^. Each swab was stored in a 1.5-ml sterile and DNase/RNase free microcentrifuge tube for ITS sequencing.

Necrotic tissue samples were collected from 32 patients who were treated with corneal ulcer debridement in a sterile operating room. Under topical anesthesia with oxybuprocaine hydrochloride eye drops, the necrotic tissue was removed using a sclerotome blade and subjected to culture after being streaked on Sabouraud dextrose agar medium.

### *In vivo* confocal microscopy

The confocal microscope HRT3 (Heidelberg Engineering, Heidelberg, Germany) was used for detecting fungal hyphae and spores with 800 magnifications. Fungal hyphae on IVCM were of bright linear, filamentous, branching or thread-like morphology, 50 to 250 μm long, in a relatively dark background (Fig. 1b), whereas spores were in an oval shape with a diameter of 10 to 15 μm (Fig. 1c)^14,15^. If any hyphae or spores were visible on images, the result of IVCM was reported as positive.

### Smear

After an addition of 10% KOH and covering with a microscopic cover glass under laminar flow, samples were detected by biomicroscopy^16^. If any hyphae or spores were observed at 400 magnifications, the result of smear was reported as positive^7^.

### Microbial culture

Corneal scrapings and necrotic tissue obtained during corneal ulcer debridement were cultured on Sabouraud dextrose agar medium under laminar flow at 25 °C and 37 °C, respectively, for 14 days, with daily observation. The result generated at 25 °C was regarded as the final result, while the 37 °C condition served for identification of dimorphic fungi^17^.

The colonies were judged by two experienced technicians according to the growth rate, color, appearance of the margin and the surface of colonies, changes during the period of culture, and difference at 25 °C and 37 °C, as well as morphology of hyphae and spores under a microscope at 400 magnifications after lactophenol cotton blue staining^16,18^. If no colony formed within 14 days, the culture was reported to be negative.

### High-throughput internal transcribed spacer sequencing

Total genomic DNA was extracted from the conjunctival sac swabs (group FD) and the corneal scrapings (group FS) using the DNA Extraction Kit (Cat. No. D3096-100T, Omega Bio-tek, Norcross, GA, USA) following the manufacturer’s instructions. Then all DNA samples were sent to Qingdao OE Biotech (Qingdao, China; https://www.qdoebiotech.com) for ITS sequencing. The kits and instruments used are shown in Supplementary Tables 1 and 2.

#### DNA amplification

After the quality and quantity of DNA were checked by NanoDrop and agarose gel, all samples were loaded into 0.6% agarose gel and subjected to 120 V constant voltage electrophoresis for 15 minutes. Totally 50 ng of DNA without degradation or with slight degradation were used for PCR amplification after being diluted to 1 ng/μl as template, with primers and Takara Ex Taq (Takara), following instructions. ITS1F primer (5′-CTTGGTCATTTAGAGGAAGTAA-3′) and ITS2 primer (5′-GCTGCGTTCTTCATCGATGC-3′) were used for amplification of fungal ITS I variable regions.

#### Library construction

Amplicon quality was demonstrated using gel electrophoresis and refined with AMPure XP beads (Agencourt), before the amplicon was enlarged for another round of PCR using ITS1F and ITS2 primers and purification with AMPure XP beads. After being quantified by using Qubit dsDNA assay kit (Cat. No. Q32854, Invitrogen, Carlsbad, CA, USA), the amplicon with a single band of 457 bp and a concentration higher than 20 ng/ul was used for sequencing. Equal amounts of purified amplicon were pooled for subsequent sequencing (Illumina miseq pe300).

#### Bioinformatic analysis

Raw sequencing data were saved as FASTQ format. Paired-end reads were preprocessed using Trimmomatic software^19^ to seek and cut out ambiguous bases (N). Trimmomatic software also cut off low quality sequences with an average quality score below 20 through the sliding window trimming approach, after which paired-end reads were assembled using FLASH software^20^. Parameters of the assembly were set as 10 bp of minimal overlapping, 20% of maximum mismatch rate, and 200 bp of maximum overlapping. Moreover, sequences were denoised by quitting reads with ambiguous, homologous sequences or those below 200 bp. Reads with 75% of bases beyond Q20 were reserved, while those having chimera were removed. The two processes were performed using QIIME software (version 1.8.0)^21^. Clean reads were further subjected to primer sequences, wiping off and clustering to form operational taxonomic units (OTUs) with 97% similarity cut out using Vsearch software. All representative reads of every OTU were chosen using QIIME package. Thereafter each representative read was annotated and blasted using the Unite database (ITSs rDNA)^22,23^ and the NCBI nucleotide database^24^. 97% similarity was employed to distinguish species.

#### Identification of the causative fungi in fungal keratitis

**Figure 3:**
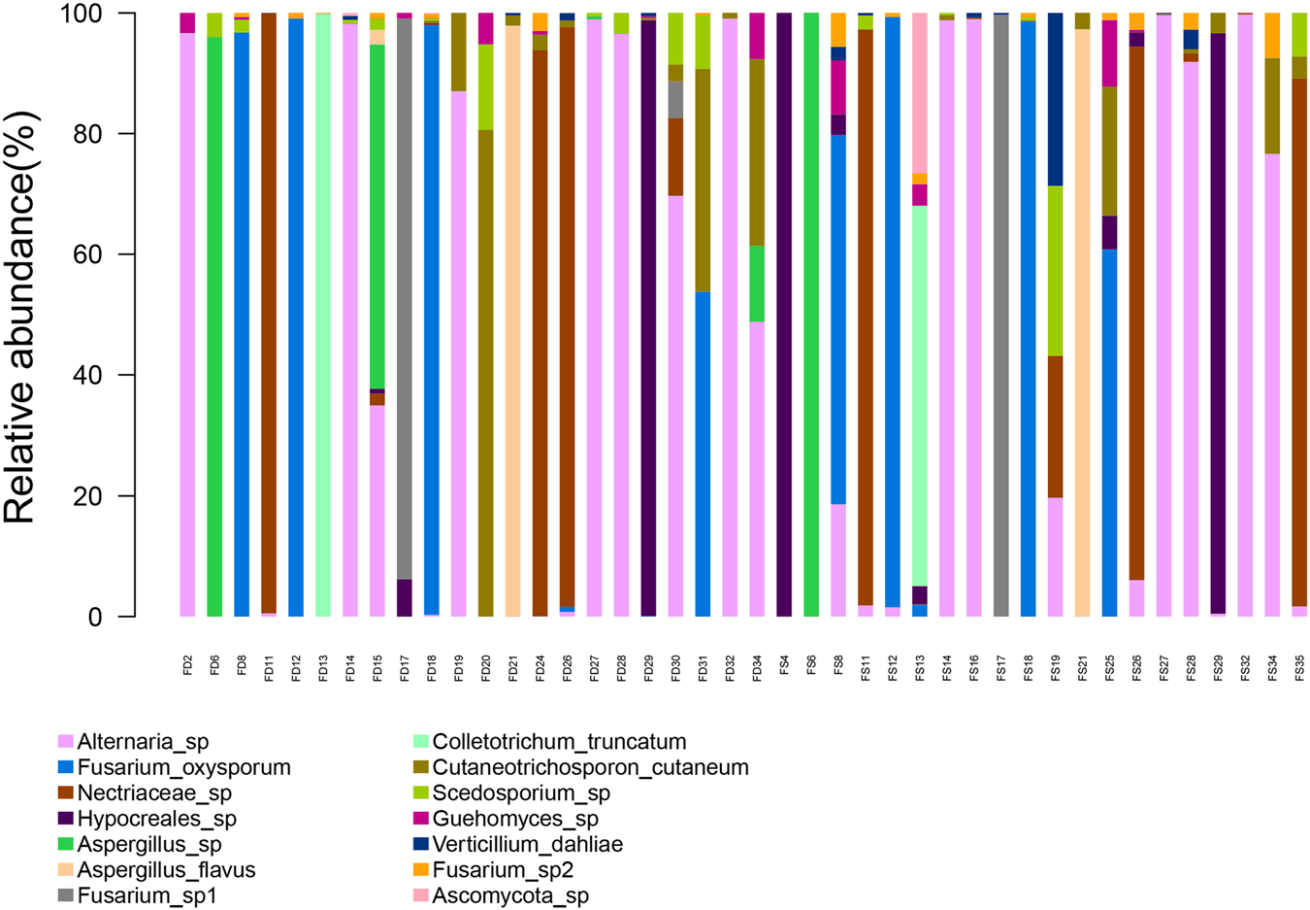
Each bar graph indicates relative quantities of different fungi in a sample. The dominant fungal genus identified by ITS sequencing was regarded as the causative pathogen of FK.

Each bar graph in Fig. 3 indicates relative quantities of different fungi in a sample. We hypothesized that the dominant fungal genus was the result of ITS sequencing for pathogen identification.

## Results

### Diagnosis of FK

**Figure 4:**
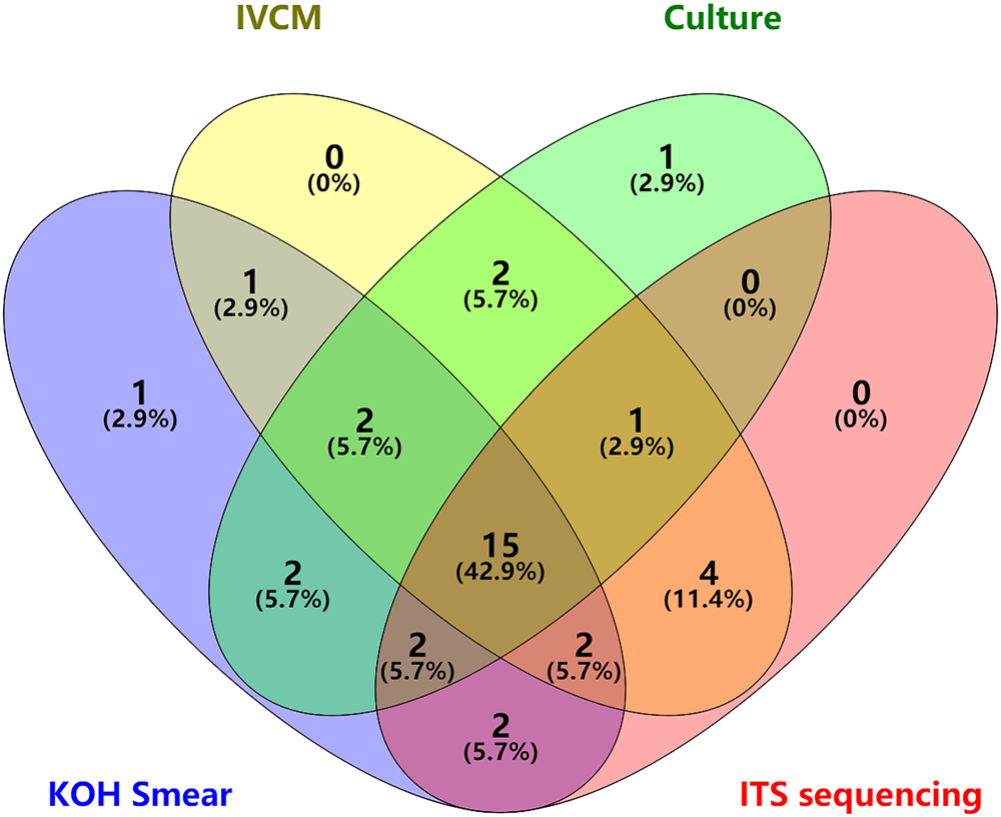
In the Venn diagram of IVCM, KOH smear, culture, and ITS sequencing, the sum of all the numbers in a same colored ellipse represents the number of patients who had a positive result by the annotated method. The number in an overlapping area of two or more ellipses represents the number of patients who were positive for two or more methods.

Thirty-five patients were eventually diagnosed with FK, with fungal pathogens found by IVCM in 27 patients (77.14%), by KOH smear in 27 patients (77.14%), and by culture in 25 patients (71.43%) (Fig. 4 and Table 1). It took an average of 6 days (3 to 13 days) to obtain the culture results. Thirty-four of the 35 patients (97.14%) were diagnosed immediately via the rapid testing approaches of IVCM or KOH smear.

**Table 1:** Results of IVCM, KOH smear, culture, and ITS sequencing.

Patients	IVCM	Smear	Culture		ITS sequencing		Consistency in results between ITS sequencing and culture		
			Corneal scraping	Necrotic tissue from corneal ulcer debridement	Conjunctival swab	Corneal scraping			
1	+	−	*Acremonium* (13 days)	*Acremonium* (11 days)	−	−	−		
2	+	+	*Alternaria* (10 days)	*Alternaria* (8 days)	*Alternaria*	−	*Y*		
3	+	+	−	−	−	−	−		
4	+	+	*Fusarium* (6 days)	*Fusarium* (7 days)	−	*Hypocreales_sp*	?		
5	+	+	*Saccharomyces* (7 days)	*Saccharomyces* (8 days)	−	−	−		
6	+	+	*Aspergillus* (6 days)	−	*Aspergillus*	*Aspergillus*	*Y*		
7	+	+	*Fusarium* (4 days)	*Fusarium* (6 days)	−	−	−		
8	+	+	*Fusarium* (7 days)	*Fusarium* (4 days)	*Fusarium oxysporum*	*Fusarium oxysporum*	*Y*		
9	−	−	*Fusarium* (8 days)	−	−	−	−		
10	+	−	*Fusarium* (8 days)	*Fusarium* (5 days)	−	−	−		
11	+	+	*Fusarium* (5 days)	*Fusarium* (5 days)	*Nectriaceae sp*	*Nectriaceae sp*	?		
12	+	+	*Alternaria* (5 days)	*Alternaria* (5 days)	*Fusarium oxysporum*	*Fusarium oxysporum*	*N*		
13	−	+	−	−	*Colletotrichum truncatum*	*Colletotrichum truncatum*	−		
14	−	+	*Alternaria* (4 days)	*Alternaria* (6 days)	*Alternaria*	*Alternaria*	*Y*		
15	+	+	*Alternaria* (4 days)	*Alternaria* (7 days)	*Aspergillus*	−	*N*		
16	−	+	−	−	−	*Alternaria*	−		
17	+	−	−	−	*Fusarium*	*Fusarium*	−		
18	+	+	−	−	*Fusarium oxysporum*	*Fusarium oxysporum*	−		
19	+	+	*Alternaria* (5 days)	*Alternaria* (7 days)	*Alternaria*	*Verticillium dahliae*	*N*		
20	+	−	−	−	*Cutaneotrichosporon cutaneum*	−	−		
21	−	+	−	−	*Aspergillus flavus*	*Aspergillus flavus*	−		
22	−	+	*Alternaria* (5 days)	*Alternaria* (6 days)	−	−	−		
23	+	−	−	−	−	−	−		
24	+	−	*Fusarium* (5 days)	*Fusarium* (5 days)	*Nectriaceae sp*	−	?		
25	+	+	*Fusarium* (5 days)	−	−	*Fusarium oxysporum*	*Y*		
26	+	+	*Fusarium* (4 days)	*Fusarium* (4 days)	*Nectriaceae sp*	*Nectriaceae sp*	?		
27	+	+	*Alternaria* (5 days)	−	*Alternaria*	*Alternaria*	*Y*		
28	+	+	*Alternaria* (6 days)	*Alternaria* (4 days)	*Alternaria*	*Alternaria*	*Y*		
29	−	+	*Alternaria* (5 days)	*Alternaria* (5 days)	*Hypocreales sp*	*Hypocreales sp*	?		
30	+	+	*Alternaria* (6 days)	*Alternaria* (5 days)	*Alternaria*	−	*Y*		
31	+	+	−	−	*Fusarium oxysporum*	−	−		
32	+	+	*Alternaria* (7 days)	/	*Alternaria*	*Alternaria*	*Y*		
33	−	+	*Aspergillus* (3 days)	/	−	−	−		
34	+	+	*Alternaria* (7 days)	*Alternaria* (6 days)	*Alternaria*	*Alternaria*	*Y*		
35	+	−	−	−	−	*Nectriaceae sp*	−		
36	−	−	−	−	−	−	−		
37	−	−	−	−	−	−	−		
38	−	−	−	−	−	−	−		
Number of samples with positive results	27	27	25	19	22	20	*10Y*	*3 N*	*5?*
Number of patients with positive results	27	27	25		26		*10Y*	*3 N*	*5?*

+: Positive result.

−: Negative result.

/: The approach was not used.

Y: Results of ITS sequencing and culture were same at the genus level.

N: Results of ITS sequencing and culture were different. ?: Results of ITS sequencing and culture may be same with integrity of the database.

### Identification of pathogenic fungi

Among the 68 cultured samples from patients diagnosed with FK, 44 (64.71%) showed positive results. No conflicting genus, for each individual patient, was found in corneal scrapings and necrotic tissue (Table 1). Furthermore, no conflicting result existed in the 25 °C and 37 °C culture conditions.

Both conjunctival sac swabs (group FD) and corneal scrapings (group FS) from each of the 35 patients were subjected for ITS sequencing, which required 7 days to obtain raw data. The quality test showed the number of samples, 22 in group FD (qualification rate 62.86% for FK patients) and 20 in group FS (qualification rate 57.14% for FK patients), was adequate for further analysis. The samples from 9 patients were qualified in neither group, while those from 16 patients were qualified in both groups, and the same dominant genus was detected from the two samples of 15 patients. Alpha diversity (P = 0.74, Shannon index, Wilcoxon rank sum test) and beta diversity (P = 0.938999, ANOSIM, Bray Curtis) showed the two groups could be substituted by each other to some extent. As a consequence, the amount of the qualified cases in the two groups both reached 26 (74.29% of FK patients).

One patient (No. 19) demonstrated different results in the two samples for ITS sequence analysis. *Alternaria* and *Verticillium dahliae* were detected in the conjunctival sac and the cornea, respectively, while the culture result presented with *Alternaria*. To avoid exaggerating the consistency between ITS sequencing and culture and considering that the corneal scraping may be more representative for the diseased area, the patient was subsequently evaluated according to the result of the corneal sample.

**Figure 5:**
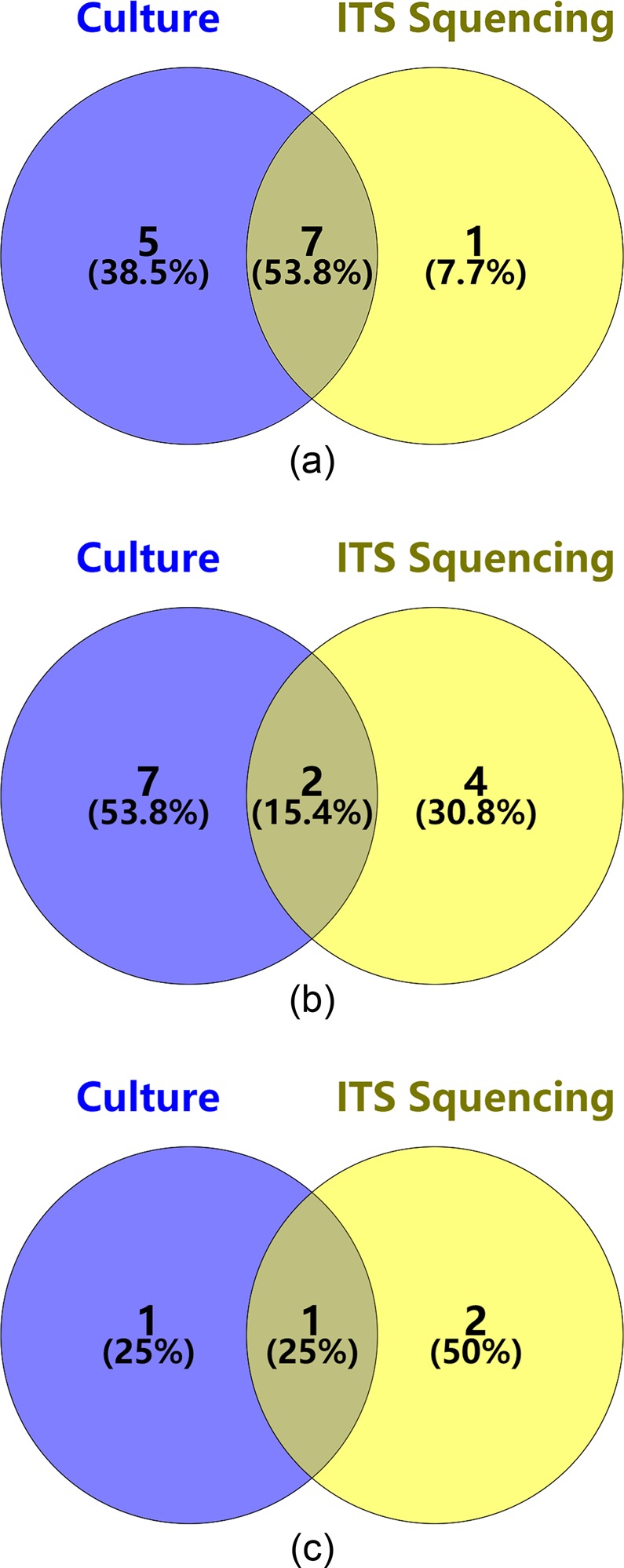
The results of culture and ITS sequencing were same in 10 patients with *Alternaria* (**a**), *Fusarium* (**b**), and *Aspergillus* (**c**) identified. The number in each overlapping area means the amount of consistent results between the two testing approaches. Different pathogenic fungi varied in consistency for both of them.

In the 26 ITS sequencing reports, there were 8 cases of *Alternaria*, 6 cases of *Fusarium*, 3 cases of *Aspergillus*, 4 cases of *Nectriaceae*, 2 cases of *Hypocreales*, 1 case of *Colletotrichum truncatum*, 1 case of *Verticillium dahlia*, and 1 case of *Cutaneotrichosporon cutaneum*. On the other hand, in the final 25 cases of positive fungal culture, there were 12 cases of *Alternaria*, 9 cases of *Fusarium*, 2 cases of *Aspergillus*, 1 case of *Saccharomyces*, and 1 case of *Acremonium*. The test results of culture and ITS sequencing were consistent in only 10 patients (28.57% of patients with FK), under the hypothesis that the dominant genus was the result of ITS sequence analysis, with *Alternaria*, *Fusarium*, and *Aspergillus* being the pathogenic fungal genera (Fig. 5).

Two of the 26 ITS sequencing reports disclosed results at the order level, 4 at the family level, 11 at the genus level, and 9 at the species level. However, all fungal culture results were at the genus level. The ITS sequencing results in 20 patients (57.14% of patients with FK) met or exceeded the genus level.

## Discussion

### Diagnostic approaches to FK

FK can be determined based on any positive result of IVCM, KOH smear or culture. However, a longer time is needed for culture compared with the former two approaches. In the current study, 34 of 38 patients (89.47%) who were suspected of having FK were diagnosed rapidly using IVCM or KOH smear. Although an early correct diagnosis can be achieved, further identification of fungal strains is important for targeted treatment. Since fungal community was reported to abound on healthy ocular surface^4^, we designed this research to evaluate the role of high-throughput ITS sequencing in identifying fungal pathogens of FK.

### Culture for identification of causative fungi

A successful antifungal chemotherapy for FK depends much on accurate identification of the genus of causative fungi^3,25^. Culture has been considered as a gold standard for the diagnosis of FK, and physicians often refer to the antifungal susceptibility of culture when selecting antifungal medication.

Different fungal genera sometimes present similar appearance, while the same fungal genus may show a series of varying morphological features. Identification of a specific fungal genus is mainly based on its morphology, but judgment might be false when colonies and spores show atypical morphology^26^. Misjudgment associated with the observer’s experience may also occur. Furthermore, fungi usually grow slowly^16^. Some fungi cannot be cultured and separated yet because of lacking knowledge about suitable culture conditions^27^. Certain previous empirical treatment can reduce fungal activity and affect culture results^28^. These factors make fungal culture complicated.

### High-throughput ITS sequencing for identification of causative fungi

High-throughput ITS sequencing possesses many advantages over culture for identification of fungal pathogens in FK. Not influenced by the activity of fungi, ITS sequence analysis requires only a small amount of DNA to be sequenced using an automated sequencer. With ITS sequence used as a universal DNA barcode marker for fungi^27,29^, this accurate molecular diagnostic technique includes DNA sequencing of ITS sequences and comparing the sequenced ITS sequences with known fungal ITS sequences to obtain fungal genus information. Moreover, ITS sequencing has high sensitivity and specificity to confirm fungal infection^30^. Relative content of almost all genera in a sample could be measured as long as the sample is qualified. The ITS sequencing reports convey not only a diagnosis but also detailed information of fungi on corneal surface. Thus, ITS sequencing may further guide scientific research with big data and bioinformatics, help to enrich the public sequencing database, and provide a new tool for discovering novel pathogenic fungi in cornea^30^.

However, this test method has limitations. First, ITS sequencing depends on the integrity of the database, but the public sequence database remains a few flaws^31^. For example, the current morphology-based taxonomic schemes neglect the phylogenetic analysis, which is more compatible with ITS sequencing^31,32^. Besides, the public sequence database includes some errors to be rectified^33^. Second, when the detected sequence does not correspond to any specific known genus, the result can only be delivered at the class, order or family level. Consequently, its significance for clinical treatment may be reduced. Third, every ITS primer has its own success rate for a range of fungi, so an appropriate solution is to use more than one primer^29^. Although we used combined primers of ITS1F and ITS2 as most other researchers did, the test results might be somewhat less than ideal. Fourth, ITS sequencing can only identify fungal pathogens of FK rather than diagnose FK. Fifth, the positive rate of ITS sequencing is not significantly improved compared with culture, partly due to the scarce quantity of collected fungi, choice of reagents and kits, and PCR bias^34^.

### Divergence between culture and ITS sequencing

Our study showed that only 28.57% of patients with FK had the same results of culture and ITS sequencing. It is a harsh phenomenon that ITS sequence analysis challenges culture, an acknowledged standard for fungal identification. The results of culture showed more *Fusarium* and *Alternaria*, when the results of ITS sequencing differed from fungal culture. It may be partly because *Fusarium* and *Alternaria* are more easily cultured on Sabouraud dextrose agar. If a variety of media are used for catering for causative fungi, the consistency of the positive rate between the two methods may tend to change. It is also noteworthy that *Hypocreales* includes *Nectriaceae*, while *Nectriaceae* includes *Fusarium*. Therefore, with the improvement of the public sequencing database, the amount of *Fusarium* in ITS sequencing reports is expected to increase.

With the development of high-throughput sequencing, we can imagine the possibility of analyzing a gigabit of data such as ITS sequences into a simpler conclusion in the future^35^. At that time, ITS sequencing may guide clinical treatment accurately^36^. But it does not mean we do not need to culture microorganisms anymore. Culture will always be essential^35,37^. Cultivation-dependent and cultivation-independent approaches not only complement each other, but in fact need each other^37^. On one hand, the current databases are imperfect, which requires information of new fungal species as detected by culture^37,38^. On the other hand, multi-omic methods including ITS sequencing can be used to predict suitable conditions for culture of hard-to-culture species on the basis of its metabolic network^37,39^.

### Spectrum of fungal keratitis

In this study, the most frequently encountered causative fungal genus was *Alternaria*, followed by *Fusarium* and *Aspergillus*, according to both culture and ITS sequencing. This seems to be different from the global spectrum, in which *Fusarium*, *Aspergillus*, and *Candida* are the most common fungal pathogens^1^.

The spectrum of FK varies in different countries. In India, *Fusarium* is the predominant causative fungal genus, followed by *Aspergillus*, *Cladosporium*, *Botryodiploidia*, and *Curvularia*^8^. In Brazil, *Fusarium*, *Aspergillus*, *Candida*, *Penicillium*, and *Cladosporium* are the major pathogenic fungi^40^. The spectrum of FK also differs in varied areas of a country. The most common causative fungal genus in north China is *Fusarium*, followed by *Aspergillus*, *Alternaria*, *Penicillium*, and *Candida*^41^. In central China, the first three genera are same with north China, but *Mycelia sterilia* and *Penicillium* rank fourth and fifth, respectively^42^.

Our series of patients are not sufficient for demonstrating the epidemiologic aspects of FK, and the spectrum of fungal genus in this study may be related to the kind of crops acclimatized to the season during the research^1^. The epidemiology of FK needs to be further investigated using cultivation-independent approaches, for the existence of discordance between culture and high-throughput ITS sequencing results.

## Conclusion

High-throughput ITS sequencing challenged fungal culture in identifying the genus and species of pathogenic fungi of FK and could generate more information about fungal microbiota on ocular surface. After weighing the pros and cons, ITS sequencing technique seems to be an optional complement for identification of fungal pathogens in the diagnosis of FK.

## Supplementary information

Table S1 and Table S2.
