# Supplementary material for: Diagnostic information Profiling and Evaluation of Causative Fungi of Fungal Keratitis Using High-throughput Internal Transcribed Spacer Sequencing
Source: Sci Rep. 2020 Feb 3;10:1640. doi: 10.1038/s41598-020-58245-7 (PMC6997210; doi:10.1038/s41598-020-58245-7)
Supplement: Supplementary file 1 — Table S1 and Table S2. [file 41598_2020_58245_MOESM1_ESM.pdf]

Title:

Diagnostic information Profiling and Evaluation of Causative Fungi of Fungal Keratitis Using High-throughput Internal Transcribed Spacer Sequencing

Keywords:

Fungal keratitis; Diagnosis; Internal Transcribed Spacer; High Throughput Sequencing

Author:

Zhichao Ren[1,2], Qing Liu[2,3], Yuqian Wang[2], Yanling Dong[2], Yusen Huang[2]

Author Affiliations:

1. School of Medicine and Life Sciences, University of Jinan-Shandong Academy of Medical Sciences, Jinan, China
2. State Key Laboratory Cultivation Base, Shandong Provincial Key Laboratory of Ophthalmology, Shandong Eye Institute, Shandong First Medical University & Shandong Academy of Medical Sciences, Qingdao, China
3. Co-first author

Correspondence to Dr. Yusen Huang, Shandong Eye Institute, 5 Yanerdao Road, Qingdao 266071, China. Email: huang_yusen@126.com

Kits Name	Producers	Cat.No.
DNeasy PowerSoil Kit(100)	QIAGEN	12888-100
QIAamp 96 PowerFecal QIAcube HT kit(5) Q	QIAGEN	51531
Qubit dsDNA Assay Kit	Invitrogen	Q32854
Takara Ex Taq	Takara	RR001Q

Table S1: Kits used for ITS sequencing

Instrument Name	Producers	Cat.No.
Centrifuge	Eppendorf	Centrifuge 5418
PCR	Bio-rad	580BR10905
QIAxtractor	QIAGEN	SN 002358
DNA Electrophoresis Cell	Tanon	HE-120
Gel Imaging System	Tanon	2500
Pipette	Eppendorf	-
Bioanalyzer	Aglient	2100
Tips, Centrifuge Tube	Axygen	-

Table S2: Instruments used for ITS sequencing
